# A randomized clinical trial of the effects of leafy green vegetables and inorganic nitrate on blood pressure

**DOI:** 10.1093/ajcn/nqaa024

**Published:** 2020-02-24

**Authors:** Michaela L Sundqvist, Filip J Larsen, Mattias Carlström, Matteo Bottai, John Pernow, Mai-Lis Hellénius, Eddie Weitzberg, Jon O Lundberg

**Affiliations:** 1 Department of Physiology and Pharmacology, Karolinska Institutet, Stockholm, Sweden; 2 Medical Unit Clinical Nutrition, Karolinska University Hospital, Stockholm, Sweden; 3 Department of Sport and Health Sciences, Swedish School of Sport and Health Sciences, Stockholm, Sweden; 4 Division of Biostatistics, Institute of Environmental Medicine, Karolinska Institutet, Stockholm, Sweden; 5 Department of Medicine, Karolinska University Hospital, Stockholm, Sweden; 6 Department of Perioperative Medicine and Intensive Care, Karolinska University Hospital, Stockholm, Sweden

**Keywords:** leafy green vegetables, dietary nitrate, nitric oxide, blood pressure, hypertension

## Abstract

**Background:**

A diet rich in fruits and vegetables is associated with lowering of blood pressure (BP), but the nutrient(s) responsible for these effects remain unclear. Research suggests that inorganic nitrate present in leafy green vegetables is converted into NO in vivo to improve cardiovascular function.

**Objective:**

In this study, we evaluated the effect of leafy green vegetables on BP in subjects with elevated BP, with the aim of elucidating if any such effect is related to their high nitrate content.

**Design:**

We enrolled 243 subjects, 50–70 y old, with a clinic systolic BP (SBP) of 130–159 mm Hg. After a 2-wk run-in period on a nitrate-restricted diet the subjects were randomly assigned to receive 1 of the following 3 interventions daily for 5 wk: low-nitrate vegetables + placebo pills, low-nitrate vegetables + nitrate pills (300 mg nitrate), or leafy green vegetables containing 300 mg nitrate + placebo pills. The primary end point measure was the difference in change in 24 h ambulatory SBP between the groups.

**Results:**

A total of 231 subjects (95%) completed the study. The insignificant change in ambulatory SBP (mean ± standard deviation) was −0.6 ± 6.2 mm Hg in the placebo group, −1.2 ± 6.8 mm Hg in the potassium nitrate group, and −0.5 ± 6.6 mm Hg in the leafy green vegetable group. There was no significant difference in change between the 3 groups.

**Conclusions:**

A 5-wk dietary supplementation with leafy green vegetables or pills containing the same amount of inorganic nitrate does not decrease ambulatory SBP in subjects with elevated BP. This trial was registered at clinicaltrials.gov as NCT02916615.

## Introduction

High blood pressure (BP) affects more than 1 billion adults worldwide and represents the leading risk factor for cardiovascular disease (1). High-normal BP is defined as a systolic BP (SBP) of 130–139 and/or diastolic BP (DBP) 85–89 mm Hg and grade 1 hypertension as SBP 140–159 mm Hg and/or DBP 90–99 mm Hg, according to guidelines from the European Society of Cardiology (2). That definition was recently reviewed by the American Heart Association, followed by updated guidelines defining stage I hypertension as SBP 130–139 and DBP 80–89 mm Hg (3). In addition to pharmacological approaches, lifestyle changes, including increasing physical activity and consuming a healthy diet, are important components of prevention and management of hypertension (4). In the Dietary Approaches to Stop Hypertension (DASH) trial, a diet rich in fruit and vegetables was shown to decrease BP in healthy as well as hypertensive subjects compared with a control diet low in fruit and vegetables (5). Also, epidemiological studies have indicated that within the vegetable food group, leafy green vegetables provide the strongest protection against cardiovascular disease (6–8). However, the active nutrient(s) responsible for these beneficial effects remains to be elucidated.

Leafy green vegetables are naturally very rich in nitrate, an inorganic anion previously claimed to have deleterious health effects (9). Therefore, in order to minimize exposure to this potentially toxic compound, the levels of nitrate in our food and drinking water are strictly controlled (10). However, more recent research has shown that when ingested nitrate is converted in blood and tissues to generate NO—a vasodilator and signaling molecule of great importance for cardiovascular health (11). As a first step in the nitrate-nitrite-NO pathway, nitrate is converted to nitrite by anaerobic bacteria in the oral cavity (12). In the acidic environment of the stomach, nitrite spontaneously forms NO, while the part remaining intact is taken up into the small intestine and enters the systemic circulation (13). Intriguingly, a number of enzymes and proteins in blood and tissues have been shown to reduce nitrite to NO (14). A substantial number of smaller trials in healthy and hypertensive subjects have now shown that nitrate has beneficial cardiovascular effects, including lowering of BP (15–25). However, a larger controlled study looking at the effects of dietary-derived nitrate on BP in subjects with elevated BP is still lacking. Moreover, most previous studies have used either a juice (often beetroot) or a nitrate salt to evaluate the effects of nitrate. However, nitrate intake in the population derives mainly from various leafy green vegetables. It would therefore be of importance to use this food group in a controlled clinical study as the results may influence dietary recommendations for this large patient group.

We hypothesized that leafy green vegetables, due to their high nitrate content, would reduce BP in hypertensive patients. The present study is a single-site, randomized, placebo controlled clinical trial comparing the effects on BP of leafy green vegetables with those of low-nitrate vegetables or a nitrate salt. Inclusion of the group receiving a nitrate salt enabled us to determine if a similar reduction in BP occurred in both nitrate groups, which would pinpoint this anion as the active ingredient.

## Methods

The study subjects were 50–70-y-old women and men with a clinical SBP of 130—159 mm Hg and DBP <110 mm Hg, recruited in Stockholm, Sweden. Exclusion criteria included a cardiovascular event within the previous 6 mo, change in dose of antihypertensive medication within the previous 2 mo, or use of organic nitrates or proton pump inhibitors. Also, persons who were vegetarian, vegan, or allergic to any of the vegetables included in the study protocol could not be enrolled. Subjects were allowed to continue taking their BP medication during the trial. A detailed list of inclusion and exclusion criteria is presented in **Supplemental Table 1**. The study was approved by the local research ethics committee in Stockholm and performed according to the declaration of Helsinki. Subjects gave their written informed consent before inclusion in the study.

### Study design

The study (registered at clinicaltrials.gov as NCT02916615) was randomized, placebo controlled, and single blinded. The investigators were unaware of the randomly assigned groups. The participants were unaware of which type of diet was rich in nitrate. The study was performed at Karolinska University Hospital, Department of Cardiology Clinical Research Unit, between September 2014 and December 2018.

The study comprised 3 phases: screening, run-in, and intervention. Subjects were recruited through advertisements in local newspapers. The screening included 1 telephone screening and 2 visits at the clinic. At each screening visit, clinic BP was measured by a biomedical analyst or dietitian with an automatic Omron M10-IT BP monitor (Omron Corporation) after 5 minutes of rest in a seated position. Clinic BP was measured 3 times and the means of the second and third readings were used to determine BP. At the second screening visit, subjects filled in questionnaires regarding physical activity, tobacco use, and alcohol consumption. They were also instructed to perform a 3-day food record using the Riksmaten method (web based), which has been described previously (26). Subjects with a clinical SBP of 130–159 mm Hg and DBP < 110 mm Hg at the second screening visit were included in the study.

In the 2-wk run-in phase all subjects were given low nitrate vegetables (carrots, bell peppers, cherry tomatoes, and sweet corn) to be consumed twice daily together with main meals and instructed to avoid all other vegetables in their diet during the entire study period. At the end of the run-in period a 24 h urine sample was collected and 24 h BP monitoring was performed using WatchBP® O3 (Microlife Corporation, Switzerland), validated by the European Society of Hypertension. The monitor was programmed for reading every 30 minutes. In the morning before the 24 h BP measurement fasting blood- and saliva samples were collected and clinic BP was measured. The urine, plasma, and saliva samples were stored at −80 C° until nitrate and nitrite were measured using a high- performance liquid chromatography (HPLC) system (ENO-20; EiCom) as previously described (27).

The intervention phase started with randomization (*n* = 9 in each block) to one of 3 groups: low nitrate vegetables + placebo pills (potassium chloride, twice daily), low nitrate vegetables + nitrate pills (150 mg nitrate in the form of potassium nitrate, twice daily) or leafy green vegetables (amount adjusted to contain 150 mg nitrate, twice daily) + placebo pills. A research nurse was responsible for the randomization protocol. The subjects were instructed to take a pill with their given vegetables 2 times daily. The groups receiving low nitrate vegetables were double blinded and the leafy green vegetables group was single blinded, i.e., the investigators knew that they were given a placebo pill. The nitrate content in the leafy green vegetables was measured 4 times each year using a chemiluminescence method described in detail earlier (28). The exact amount of vegetables given to the patients was adjusted so as to ensure a daily intake of 300 mg (2 × 150 mg) nitrate. The amount (weight) of vegetables was matched in the 3 groups. Vegetables were delivered from a local grocery store during the whole study period. The intervention phase lasted 5 wk with weekly visits at the hospital for clinic BP and to collect the weekly quantity of vegetables. Subjects reported weekly if vegetable or pill intake had not been according to protocol. In the last week of the intervention phase 24-h urine collection, 24-h BP monitoring, and blood and saliva sampling, as well as the questionnaires and 3-day food record were repeated.

Flow mediated dilation (FMD) was performed because intake of nitrate has previously been shown to affect this cardiovascular parameter (29–31). In subgroups from the 3 interventions (*n* = 14–15 in each group), FMD was performed at baseline and after interventions. The brachial artery of the nondominant arm was used for determination of FMD. A cuff was placed around the upper forearm and inflated to 30 mm Hg above SBP or 200 mm Hg for 5 min. After deflation of the cuff, the diameter of the brachial artery was recorded for 3 min during hyperemia. FMD was calculated as a percentage increase in diameter from baseline. A detailed description of the method has been published previously (32).

### Outcomes

The primary outcome measure was the difference in change in 24 h SBP between the intervention groups. A change in BP was the difference between BP before and after the intervention period. Secondary outcome measures were the differences in change in 24 h DBP, clinic BP, ambulatory BP during daytime (08:00 to 22:00) and night-time (22:00 to 08:00) and FMD.

### Statistical analysis

The Dietary Nitric Oxide (DINO) study was designed to investigate if a daily intake of leafy green vegetables lowers BP in people with elevated BP and to test the following hypothesis: 300 mg nitrate provided by leafy green vegetables or pills will lower 24 h SBP compared with intake of low nitrate vegetables and placebo pills. To achieve balance in the allocation to the 3 groups, a block design for random assignment was made by a statistician. The software used for random assignment was Stata version 13 (StataCorp), and the number generator was KISS. Each block contained 9 observations, 3 per treatment arm. A prestudy power calculation was performed and a sample size of 60 subjects per group was estimated to provide > 80% power to detect a 3–mm Hg difference in change in SBP among groups by using unpaired comparisons. SDs used were estimated from the largest study at the time on the effects of nitrate on 24-h SBP in hypertensive patients (16). The assumptions for ANOVA were tested. The D'Agostino–Pearson omnibus normality test was used to test for normality. Between-group differences in baseline data, change in BP, and FMD were analyzed with 1-factor ANOVA followed by Bonferroni post hoc analysis. Fisher's exact test was used for nominal baseline data. The level of the significance was set at *P* < 0.05. Change in BP was not included if postintervention data were missing. Within-group comparison was analyzed with 2-factor repeated measures ANOVA followed by Bonferroni post-hoc analysis. Weekly SBP and DBP were analyzed with a linear mixed model. The models contained indicator variables for the treatment groups, indicator variables for the visits, interaction terms between groups and visits, and a normally-distributed random intercept for each subject.

Statistical analyses were performed with Stata version 15 (StataCorp) and PRISM 6 software (Graph Pad). All data are presented as means ± SDs.

## Results

### Baseline and descriptive data

**FIGURE 1:**
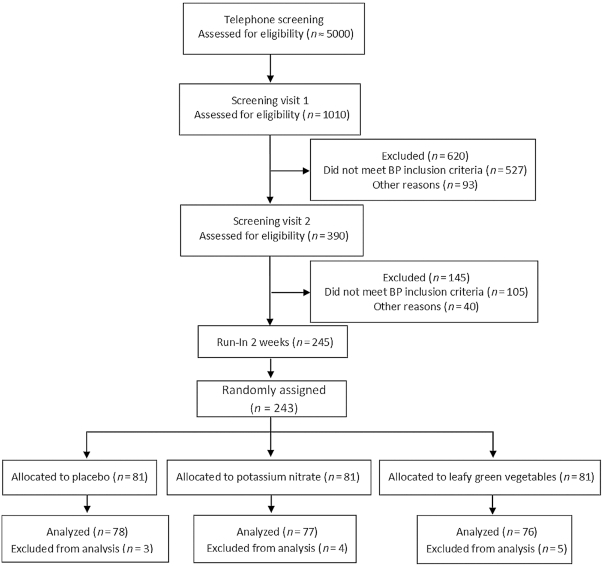
Participant flow chart. Reasons for exclusion during the intervention were increasing BP (*n* = 1), swelling and flushing in the face (*n* = 1), not wanting to continue (*n* = 3), diarrhea (*n* = 3), and starting new medication during the study (*n* = 4). BP, blood pressure.

A total of 1010 subjects were initially screened for BP and 243 were randomly assigned after completion of the 2 screening visits and a 2-wk run-in period. Of these, 231 completed the study (122 women and 109 men). Baseline characteristics are described in **Table 1** and a participant flow chart is presented in **Figure 1**.

**TABLE 1:** Characteristics of the subjects in the 3 study groups^1^

	Group		
	Placebo (*n* = 78)	Potassium nitrate (*n* = 77)	Leafy green vegetables (*n* = 76)
Age, y	63 ± 5.6	62 ± 5.5	63 ± 5.2
Females, *n*	42	38	42
Weight, kg	75.8 ± 14.2	79.5 ± 13.9	76.8 ± 13.5
BMI, kg/m^2^	25.4 ± 3.6	26.6 ± 3.6	26.1 ± 3.1
Physical activity, min/wk			
Low-level exercise (walks, cycling, gardening)	395 ± 332	375 ± 298	431 ± 320
Intensive exercise (running, gymnastics, ball sports)	150 ± 113	165 ± 163	117 ± 73
Daily tobacco use, *n*	10	5	9
Excessive alcohol consumption,^2^*n*	5	1	3
Energy intake,^3^ kcal/d	2102 ± 654	1958 ± 514	1833 ± 703
Vegetable intake,^3^ g/d	183 ± 125	145 ± 77	180 ± 108
Ambulatory BP, mm Hg			
SBP	130.9 ± 9.7	133.2 ± 10.9	129.8 ± 10.3
DBP	78.0 ± 6.4	79.4 ± 7.1	77.9 ± 7.2
PP	52.9 ± 7.7	53.7 ± 7.5	52.0 ± 7.3
HR, beats/min	68.3 ± 8.3	67.7 ± 8.1	68.3 ± 8.9
BP medication, *n*	34	30	39
≥2 BP medications, *n*	8	8	15

1 Values are presented as means ± SDs unless otherwise indicated. Nominal data were analyzed with Fisher's exact test and continuous normally distributed data with 1-factor ANOVA. There were no significant group differences at baseline. BP, blood pressure; DBP, diastolic blood pressure; HR, heart rate; PP, pulse pressure; SBP, systolic blood pressure.

2 Females ≥10 and males ≥15 standard drinks/wk.

3 Based on a 3-d food record; *n* = 35, 34, and 35, respectively.

### Adherence

The amount of vegetables given to ensure a daily intake of 300 mg nitrate (2 × 150 mg) in the leafy green vegetables group varied between 98 and 184 g (mean 123.9 ± 24.8 g). The self-reported compliance to the vegetable and pill intake was >97% for vegetables and > 98% for pills in all groups.

### Plasma and urinary nitrate and nitrite

Detailed information of nitrate and nitrite in plasma, urine, and saliva are presented in **Table 2**, and other laboratory data are presented in **Supplemental Table 2**. Baseline nitrate and nitrite in plasma and urine were similar in all 3 intervention groups. Plasma nitrate increased significantly in the potassium nitrate group and in the leafy green vegetables group after the intervention (*P* < 0.0001) and remained unchanged in the placebo group. (*P* > 0.99). Plasma nitrite concentrations were significantly increased only in the potassium nitrate group (*P* = 0.048) and not in the leafy green vegetables group (*P* = 0.115). Consistent with the plasma nitrate, 24-h urinary excretion of nitrate increased (*P* < 0.0001) after intervention in the potassium nitrate and leafy green vegetables groups but was unchanged in the placebo group (*P* > 0.99). Excreted nitrate corresponded well with the given dose of nitrate in the potassium nitrate group and leafy green vegetables group (254 and 303 mg, respectively), consistent with the endogenous recycling of bioactive NO and nitrite back to nitrate. Salivary nitrate and nitrite were also increased significantly (*P* < 0.0001) in the groups receiving nitrate and remained unchanged in the placebo group. Postintervention effects on plasma, saliva, and urine nitrate and nitrite are presented in **Supplemental Figure 1**.

**TABLE 2:** BP and nitrate and nitrite concentrations in plasma, saliva, and urine before and after interventions^1^

	Group					
	Placebo		Potassium nitrate		Leafy green vegetables	
	Pre	Post	Pre	Post	Pre	Post
Nitrate						
Plasma, µmol/L	31.2 ± 18.9	32.0 ± 20.2	30.6 ± 12.7	104.8 ± 52.4^4^	30.4 ± 11.6	113.6 ± 57.9^5^
Saliva, µmol/L	285.7 ± 449	314.5 ± 596	412.4 ± 432	1802 ± 1633^5^	321.6 ± 353	1557 ± 1463^5^
Urine, µmol/L	512.5 ± 296	526.2 ± 320	581.6 ± 291	2593 ± 1175^5^	525.9 ± 297	2843 ± 1167^5^
Excreted, mg/d	51.3 ± 23.1	53.0 ± 43.5	58.1 ± 30.1	253.6 ± 86.7^5^	54.0 ± 29.7	302.5 ± 127.0^5^
Nitrite, µmol/L						
Plasma	0.34 ± 0.25	0.33 ± 0.28	0.36 ± 0.22	0.46 ± 0.38^2^	0.39 ± 0.33	0.48 ± 0.45
Saliva	147.1 ± 192	157.7 ± 160	189.6 ± 185	604.0 ± 633^5^	185.7 ± 214	498.5 ± 648^5^
24-h ambulatory BP, mm Hg						
SBP	130.9 ± 9.7	130.4 ± 9.9	133.2 ± 10.9	132.0 ± 11.2	129.8 ± 10.3	129.3 ± 10.8
DBP	78.0 ± 6.4	78.5 ± 7.4	79.4 ± 7.1	78.6 ± 7.3	77.9 ± 7.2	78.3 ± 7.3
PP	52.9 ± 7.7	51.9 ± 6.7	53.7 ± 7.5	53.4 ± 7.0	52.0 ± 7.3	51.0 ± 7.5
HR, beats/min	68.3 ± 8.3	69.7 ± 8.6	67.8 ± 8.1	67.8 ± 7.7	68.3 ± 8.9	68.6 ± 9.5
Daytime ambulatory BP (08:00–20:00), mm Hg						
SBP	136.7 ± 9.9	135.3 ± 9.8	138.0 ± 11.5	137.4 ± 11.7	135.0 ± 10.5	134.9 ± 11.5
DBP	82.4 ± 6.9	82.4 ± 7.7	83.4 ± 7.4	82.5 ± 7.7	82.0 ± 7.6	82.5 ± 8.3
PP	54.4 ± 8.3	52.9 ± 7.0^2^	54.6 ± 7.9	54.7 ± 7.7	53.1 ± 7.6	52.3 ± 7.7
HR, beats/min	71.5 ± 8.9	72.9 ± 9.21	70.7 ± 8.7	70.5 ± 8.1	71.0 ± 9.6	71.4 ± 9.8
Night-time ambulatory BP (20:00-08:00)						
SBP, mm Hg	119.3 ± 11.3	120.4 ± 12.6	123.1 ± 11.7	120.8 ± 12.4	118.9 ± 13.2	118.5 ± 12.3
DBP, mm Hg	69.2 ± 7.1	70.1 ± 9.2	71.2 ± 8.1	70.0 ± 8.2	69.5 ± 8.1	69.8 ± 7.4
PP	50.1 ± 7.8	50.3 ± 7.4	51.9 ± 7.2	50.8 ± 7.1	49.5 ± 8.3	48.9 ± 8.3
HR, beats/min	62.1 ± 8.4	63.1 ± 8.5	61.6 ± 8.2	61.9 ± 8.8	62.5 ± 8.5	63.1 ± 10.2
Clinic BP, mm Hg						
SBP	135.1 ± 10.4	133.9 ± 10.2	139.5 ± 11.9	135.2 ± 11.1^3^	133.7 ± 10.1	131.9 ± 9.6
DBP	88.8 ± 7.9	88.9 ± 8.6	91.4 ± 8.0	90.3 ± 8.6	88.2 ± 8.3	88.2 ± 7.2
PP	46.4 ± 9.0	45.0 ± 8.5	48.2 ± 9.6	44.9 ± 8.9^4^	44.9 ± 8.9	43.8 ± 8.1
HR, beats/min	67.2 ± 9.2	68.3 ± 11.1	68.3 ± 10.4	68.2 ± 9.1	66.5 ± 10.1	65.3 ± 10.5

1 All values are presented as means ± SDs. Within-group comparison were analyzed with repeated measures 2-factor ANOVA followed by Bonferroni post hoc analysis. BP, blood pressure; DBP, diastolic blood pressure; HR, heart rate; PP, pulse pressure; Post, postintervention values; Pre, preintervention values; SBP, systolic blood pressure.

2 Significantly different from pre value, *P* < 0.05.

3 Significantly different from pre value, *P* < 0.01.

4 Significantly different from pre value, *P* < 0.001.

5 Significantly different from pre value, *P* < 0.0001.

### Blood pressure

**FIGURE 2:**
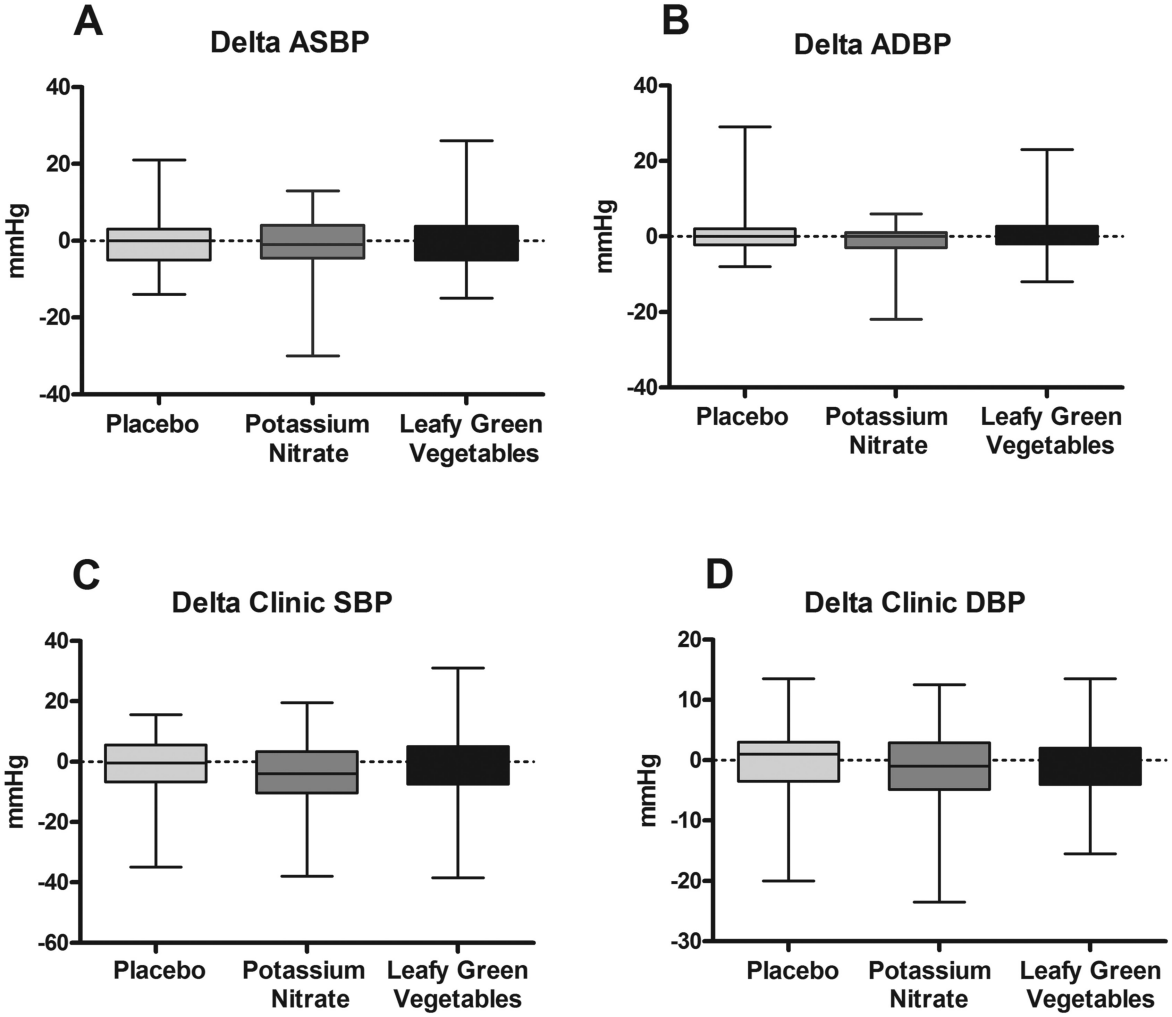
Difference in change in 24-h ambulatory systolic BP (A and B) and clinic BP (C and D) after a 5-wk intervention period. The placebo group (*n* = 78) received low-nitrate vegetables and placebo pills, the potassium nitrate group (*n* = 77) received low-nitrate vegetables and 300 mg nitrate through potassium nitrate pills, and the leafy green vegetables (*n* = 76) group received 300 mg nitrate through nitrate-rich vegetables and a placebo pill. Statistical analysis was performed with 1-factor ANOVA. There was no difference in change between the groups in 24-h ambulatory or clinic BP. All values are presented as means ± SDs. ASBP, ambulatory systolic blood pressure; BP, blood pressure.

Differences in changes between the groups in 24-h ambulatory and clinic SBP and DBP are presented in **Figure 2**. The changes in 24-h SBP after intervention were −0.6 mm Hg in the placebo group, −1.2 mm Hg in the potassium nitrate group, and −0.5 mm Hg in the leafy green vegetables group, and the changes in the 24-h DBP in these groups were 0.4, −0.8, and 0.4 mm Hg, respectively. Between-group comparisons with 1-factor ANOVA showed that there were no significant differences in changes in ambulatory SBP (*P* = 0.762) or ambulatory DBP (*P* = 0.145). Changes in clinic SBP were −1.3 mm Hg for the placebo group, −4.3 mm Hg for the potassium nitrate group, and −1.7 mm Hg for the leafy green vegetables group. Changes in clinic DBP in these groups were 0.2 mm Hg, −1.1 mm Hg, and −0.7 mm Hg, respectively. Ambulatory 24-h BP and clinic BP before and after intervention are presented in Table 2. In the potassium nitrate group there was a significant decrease in clinic SBP (*P* = 0.002) after the intervention. There was no significant difference between the groups in weekly clinic BP during the 5-wk intervention (95% CI: −7.78, 12.45; *P* = 0.650) (**Supplemental Figure 2** and **Supplemental Table 3**).

### Flow-mediated dilation

Complete FMD results are presented in **Supplemental Figure 3**. FMD at baseline was 3.4 ± 3.3% in the placebo group, 4.2 ± 3.6% in the potassium nitrate group, and 4.1 ± 3.2% in the leafy green vegetables group, and FMD was unchanged (*P* < 0.05) in all groups after the intervention period, with postintervention values of 4.7 ± 2.1%, 4.2 ± 3.5%, and 3.2 ± 3.0%, respectively.

## Discussion

In the DINO study, we investigated if leafy green vegetables or 300 mg nitrate via intake of potassium nitrate lowered 24-h ambulatory SBP in subjects with elevated BP. The protocol was well tolerated by study participants, and the drop-out rate was low (<5%). Analysis of plasma nitrate and urinary nitrate excretion confirmed excellent compliance and showed that nitrate intake and systemic uptake were nearly identical in the 2 groups receiving supplemental nitrate. If there had been a similar effect on BP in the 2 groups, the exact matching of the nitrate content in the pills to the content in the vegetables would have enabled us to pinpoint nitrate as the responsible substance. However, we found that the 24-h ambulatory BP did not change significantly in any of the 3 groups during the intervention, suggesting that the dose of nitrate achievable through normal intake of vegetables is insufficient to affect this cardiovascular parameter. A broader conclusion from these data is that intake of leafy green vegetables does not provide any reduction in BP compared with a mixture of other commonly consumed vegetables.

The dose of 300 mg nitrate per day was carefully chosen, with the main focus on what is readily achievable from a normal diet. We were also aiming for a daily overall vegetable intake close to what is seen in the general population. To achieve this the subjects were given a daily total of 98–184 g leafy green vegetables or corresponding low-nitrate vegetables, which is close to the average overall intake of vegetables in Sweden (169 and 182 g for men and women, respectively) (33). Indeed, mean reported intake of vegetables at baseline in this study population (Table 1) was in the same range. However, the amount of nitrate achievable from vegetables in a nonprocessed form did not affect ambulatory SBP. The result of this study conflicts with the effects of a number of smaller trials that have shown effects of nitrate on BP in both healthy volunteers and hypertensive patients (15–25). Typically, nitrate has been given in the form of a vegetable juice or as a salt. In a placebo-controlled study by Kapil and colleagues in 64 hypertensive subjects, supplementation with beetroot juice containing 340 mg nitrate daily for 4 wk led to a reduction (−7.7 mm Hg) in 24-h ambulatory SBP (16). In a more recent crossover study (*n* = 27) in a comparable patient group supplemented with 800 mg nitrate per d, again via beetroot juice, a reduction in 24-h SBP was not reproduced (34). In the later study, the nitrate dose was more than twice the dose used by Kapil and colleagues, which may indicate that there is a range in which nitrate is effective, with both too high and too low nitrate doses giving no effect. In addition, the study using a higher dose was only 1 wk in duration, which may have been too short to see effects.

A possible mechanism for a lack of an effect with a high dose of nitrate could be the existence of a crosstalk, i.e., formation of nitrogen oxides from the nitrate interfering with endogenous vascular NO generation from eNOS. Indeed, this phenomenon has been described in rodents given high doses of nitrate in their drinking water (35). Another reason for discrepancies between different studies regarding nitrate effects could be related to the different sources of nitrate used. In the current study, we used leafy green vegetables and sodium nitrate pills, whereas in many other studies, including the positive study by Kapil et al. mentioned above, beetroot juice was used as the nitrate source. Theoretically, there might be an additive effect of nitrate and some other compound in beetroot juice, or alternatively, the opposite situation—some compound in leafy green vegetables that attenuates the nitrate effects. However, although we cannot fully exclude these explanations, they still seem unlikely given the fact that we used potassium nitrate alone in 1 group and still saw no measurable effect in ambulatory BP.

There are some other possible reasons why nitrate did not lower BP in the current study. First, the chosen dose (300 mg) of nitrate might have been too low, although 240 mg has been reported as a threshold for a clinically relevant BP-lowering effect of nitrate (24). Second, it is possible that nitrate would have had an effect in a population with higher BP, although effect size was not found to be associated with baseline BP values in a recently published meta-analysis (36). On the other hand, the authors of this analysis argued that nitrate appears to be less effective to reduce SBP in people >60 y of age (36). A third reason might have been inefficient bioactivation of nitrate, which includes reduction of nitrate to nitrite by oral bacteria followed by further metabolism of nitrite to NO and other nitrogen oxides in the stomach (37) and in blood and tissues (11). The latter is unlikely, however, since we did control for all parameters known to affect nitrate bioactivation, including use of antibacterial mouthwash (38–39), antibiotics, proton pump inhibitors, organic nitrates, and urate-lowering drugs. Moreover, salivary concentrations of nitrite increased greatly in the nitrate groups, demonstrating effective bacterial nitrate reduction. Plasma nitrate showed significant and similar increases in the groups receiving nitrate, but plasma nitrite was significantly elevated only in the potassium nitrate group while being nearly significantly increased in the subjects receiving high-nitrate vegetables. This finding is not particularly surprising, however, considering that plasma samples were taken after overnight fasting and thereby long after the peak in plasma nitrite concentrations that follows nitrate intake. At this time point vascular NO synthase likely represents the dominant source of circulating nitrite. Finally, we cannot fully exclude that the study might have been underpowered to detect subtle differences between the groups. Even though the number of participants exceeded the sample size that was calculated in the prestudy power analysis, our data in the actual study varied more than the data in the analysis. However, the within-group numerical changes and between-group values for 24-h SBP (primary outcome measure) did not exceed 1.2 mm Hg. A difference, even had it been statistically significant in larger study groups, would still be of minor clinical importance.

The strengths of our trial include what is to our knowledge the largest group of subjects tested to date, and they were followed for a 5-wk period preceded by a 2-wk run-in period before random assignment to study groups. Moreover, the matching of the nitrate dose in subjects receiving the high-nitrate vegetables and those receiving the potassium nitrate pill enabled us to pinpoint the contribution from inorganic nitrate to any observed effects on BP. Also, by giving every subject a pill (KCl or KNO_3_), we were able to compare the results in a blinded way. The weekly visits by study participants to a single-site research clinic where they underwent BP measurements and received a collection of new fresh vegetables likely contributed to the excellent compliance and low drop-out rates. Also, the combination of 24-h ABP measurements with weekly office measurements makes overall interpretation of BP data maximally reliable.

In conclusion, our results show that intake of 300 mg nitrate daily via either leafy green vegetables or a nitrate supplement for 5 wk does not lower BP in subjects with high-normal and grade I hypertension compared with intake of a control diet low in nitrate.

## Supplementary Material

nqaa024_Supplemental_File

## Notes

Supported by grants from the Af Jochnick Foundation, the Swedish Heart-Lung Foundation, the Swedish Research Council, and ALF, and funds from the Karolinska Institutet.

EW and JOL shared senior authorship.

Supplemental Tables 1–3 and Supplemental Figures 1–3 are available from the “Supplementary data” link in the online posting of the article and from the same link in the online table of contents at https://academic.oup.com/ajcn/.

Abbreviations used: BP, blood pressure; DASH, Dietary Approaches to Stop Hypertension; DBP, diastolic blood pressure; DINO, dietary nitric oxide; FMD, flow-mediated dilation; HR, heart rate; PP, pulse pressure; SBP, systolic blood pressure.

## References

[1] WHO. Global Status Report on Noncommunicable Diseases 2014. Geneva: WHO; 2014.

[2] Williams B, Mancia G, Spiering W, Agabati Rosei E, Azizi M, Burnier M, Clement DL, Coca A, Simone G, Dominiczak A et al. 2018 ESC/ESH guidelines for the management of arterial hypertension. Eur Heart J. 2018;39:3021–104. 30165516 10.1093/eurheartj/ehy339

[3] Whelton PK, Carey RM, Aronow WS, Casey DE Jr, Collins KJ, Dennison Himmelfarb C, DePalma SM, Gidding S, Jamerson KA, Jones KA et al. 2017 ACC/AHA/AAPA/ABC/ACPM/AGS/APhA/ASH/ASPC/NMA/PCNA Guideline for the Prevention, Detection, Evaluation, and Management of High Blood Pressure in Adults: Executive Summary: A Report of the American College of Cardiology/American Heart Association Task Force on Clinical Practice Guidelines. J Am Coll Cardiol. 2018;71(19):2199–269.29146533

[4] Piepoli MF, Hoes AW, Agewall S, Albus C, Brotons C, Catapano AL, Cooney MT, Corra U, Cosyns B, Deaton C et al. 2016 European Guidelines on Cardiovascular Disease Prevention in Clinical Practice: the Sixth Joint Task Force of the European Society of Cardiology and other societies on cardiovascular disease prevention in clinical practice (constituted by representatives of 10 societies and by invited experts) developed with the special contribution of the European Association for Cardiovascular Prevention & Rehabilitation (EACPR). Atherosclerosis. 2016;252:207–74. 27664503 10.1016/j.atherosclerosis.2016.05.037

[5] Appel LJ, Moore TJ, Obarzanek E, Vollmer WM, Svetkey LP, Sacks FM, Bray GA, Vogt TM, Cutler JA, Windhauser MM et al. A clinical trial of the effects of dietary patterns on blood pressure. DASH Collaborative Research Group. N Engl J Med. 1997;336:1117–24. 9099655 10.1056/NEJM199704173361601

[6] Joshipura KJ, Ascherio A, Manson JE, Stampfer MJ, Rimm EB, Speizer FE, Hennekens CH, Spiegelman D, Willet WC Fruit and vegetable intake in relation to risk of ischemic stroke. JAMA. 1999;282:1233–9. 10517425 10.1001/jama.282.13.1233

[7] Larsson SC, Virtamo J, Wolk A Total and specific fruit and vegetable consumption and risk of stroke: a prospective study. Atherosclerosis. 2013;227:147–52. 23294925 10.1016/j.atherosclerosis.2012.12.022

[8] Hung HC, Joshipura KJ, Jiang R, Hu FB, Hunter D, Smith-Warner SA, Colditz GA, Rosner B, Spiegelman D, Willett WC Fruit and vegetable intake and risk of major chronic disease. J Natl Cancer Inst. 2004;96:1577–84. 15523086 10.1093/jnci/djh296

[9] Tannenbaum SR, Correa P Nitrate and gastric cancer risks. Nature. 1985;317:675–6. 4058577 10.1038/317675b0

[10] European Food Safety Authority. Nitrate in vegetables—Scientific Opinion of the Panel on Contaminants in the Food Chain. EFSA J. 2008;689:1–79. 10.2903/j.efsa.2008.653 PMC10193653 37213838

[11] Lundberg JO, Gladwin MT, Weitzberg E Strategies to increase nitric oxide signalling in cardiovascular disease. Nat Rev Drug Discov. 2015;14:623–41. 26265312 10.1038/nrd4623

[12] Lundberg JO, Weitzberg E, Cole JA, Benjamin N Nitrate, bacteria and human health. Nat Rev Microbiol. 2004;2(7):593–602. 15197394 10.1038/nrmicro929

[13] Lundberg JO, Govoni M. Inorganic nitrate is a possible source for systemic generation of nitric oxide. Free Radic Biol Med. 2004;37(3):395–400. 15223073 10.1016/j.freeradbiomed.2004.04.027

[14] Lundberg JO, Weitzberg E, Gladwin MT The nitrate-nitrite-nitric oxide pathway in physiology and therapeutics. Nat Rev Drug Discov. 2008;7(2):156–67. 18167491 10.1038/nrd2466

[15] Larsen FJ, Ekblom B, Sahlin K, Lundberg JO, Weitzberg E Effects of dietary nitrate on blood pressure in healthy volunteers. N Engl J Med. 2006;355:2792–3. 17192551 10.1056/NEJMc062800

[16] Kapil V, Khambata RS, Robertson A, Caulfield MJ, Ahluwalia A Dietary nitrate provides sustained blood pressure lowering in hypertensive patients: a randomized, phase 2, double-blind, placebo-controlled study. Hypertension. 2015;65:320–7. 25421976 10.1161/HYPERTENSIONAHA.114.04675 PMC4288952

[17] Webb AJ, Patel N, Loukogeorgakis S, Okorie M, Aboud Z, Misra S, Rashid R, Miall P, Deanfield J, Benjamin N et al. Acute blood pressure lowering, vasoprotective, and antiplatelet properties of dietary nitrate via bioconversion to nitrite. Hypertension. 2008;51:784–90. 18250365 10.1161/HYPERTENSIONAHA.107.103523 PMC2839282

[18] Jonvik KL, Nyakayiru J, Pinckaers PJ, Senden JM, van Loon LJ, Verdijk LB Nitrate-rich vegetables increase plasma nitrate and nitrite concentrations and lower blood pressure in healthy adults. J Nutr. 2016;146:986–93. 27075914 10.3945/jn.116.229807

[19] Hobbs DA, Kaffa N, George TW, Methven L, Lovegrove JA Blood pressure-lowering effects of beetroot juice and novel beetroot-enriched bread products in normotensive male subjects. Br J Nutr. 2012;108:2066–74. 22414688 10.1017/S0007114512000190

[20] Kerley CP, Dolan E, James PE, Cormican L Dietary nitrate lowers ambulatory blood pressure in treated, uncontrolled hypertension: a 7-d, double-blind, randomised, placebo-controlled, cross-over trial. Br J Nutr. 2018;119:658–63. 29553033 10.1017/S0007114518000144

[21] Ashworth A, Mitchell K, Blackwell JR, Vanhatalo A, Jones AM High-nitrate vegetable diet increases plasma nitrate and nitrite concentrations and reduces blood pressure in healthy women. Public Health Nutr. 2015;18:2669–78. 25683748 10.1017/S1368980015000038 PMC10271341

[22] Vanhatalo A, Bailey SJ, Blackwell JR, DiMenna FJ, Pavey TG, Wilkerson DP, Benjamin N, Winyard PG, Jones AM Acute and chronic effects of dietary nitrate supplementation on blood pressure and the physiological responses to moderate-intensity and incremental exercise. Am J Physiol Regul Integr Comp Physiol. 2010;299:R1121–31. 20702806 10.1152/ajpregu.00206.2010

[23] Jajja A, Sutyarjoko A, Lara J, Rennie K, Brandt K, Qadir O, Siervo M Beetroot supplementation lowers daily systolic blood pressure in older, overweight subjects. Nutr Res. 2014;34:868–75. 25294299 10.1016/j.nutres.2014.09.007

[24] Kapil V, Milsom AB, Okorie M, Maleki-Toyerkani S, Akram F, Rehman F, Arghandawi S, Pearl V, Benjamin N, Loukogeorgakis S et al. Inorganic nitrate supplementation lowers blood pressure in humans: role for nitrite-derived NO. Hypertension. 2010;56:274–81. 20585108 10.1161/HYPERTENSIONAHA.110.153536

[25] Lee JS, Stebbins CL, Jung E, Nho H, Kim JK, Chang MJ, Choi HM Effects of chronic dietary nitrate supplementation on the hemodynamic response to dynamic exercise. Am J Physiol Regul Integr Comp Physiol. 2015;309:R459–66. 26084693 10.1152/ajpregu.00099.2015

[26] Nybacka S, Forslund HB, Wirfalt E, Larsson I, Ericson U, Warensjö-Lemming E, Bergström G, Hedblad B, Winkvisk A, Lindroos AK Comparison of a web-based food record tool and a food-frequency questionnaire and objective validation using the doubly labelled water technique in a Swedish middle-aged population. J Nutr Sci. 2016;5:1–11. 10.1017/jns.2016.29 PMC5048186 27752306

[27] Govoni M, Jansson EÅ, Weitzberg E, Lundberg JO The increase in plasma nitrite after a dietary nitrate load is markedly attenuated by an antibacterial mouthwash. Nitric Oxide. 2008;19:333–7. 18793740 10.1016/j.niox.2008.08.003

[28] Lundberg JO, Govoni M. Inorganic nitrate is a possible source for systemic generation of nitric oxide. Free Radic Biol Med. 2004;37:395–400. 15223073 10.1016/j.freeradbiomed.2004.04.027

[29] Kleinbongard P, Dejam A, Lauer T, Jax T, Kerber S, Gharini P, Balzer J, Zotz RB, Scharf RE, Willers R et al. Plasma nitrite concentrations reflect the degree of endothelial dysfunction in humans. Free Radic Biol Med. 2006;40:295–302. 16413411 10.1016/j.freeradbiomed.2005.08.025

[30] Rodriguez-Mateos A, Hezel M, Aydin H, Kelm M, Lundberg JO, Weitzberg E, Spencer JP, Heiss C Interactions between cocoa flavanols and inorganic nitrate: additive effects on endothelial function at achievable dietary amounts. Free Radic Biol Med. 2015;80:121–8. 25530151 10.1016/j.freeradbiomed.2014.12.009

[31] Lara J, Ashor AW, Oggioni C, Ahluwalia A, Mathers JC, Siervo M Effects of inorganic nitrate and beetroot supplementation on endothelial function: a systematic review and meta-analysis. Eur J Nutr. 2016;55:451–9. 25764393 10.1007/s00394-015-0872-7

[32] Kovamees O, Shemyakin A, Pernow J Effect of arginase inhibition on ischemia-reperfusion injury in patients with coronary artery disease with and without diabetes mellitus. PLoS One. 2014;9:e103260. 25072937 10.1371/journal.pone.0103260 PMC4114552

[33] Livsmedelsverket. Riksmaten 2010–11, Livsmedels-och näringsintag bland vuxna i Sverige [Riksmaten 2010–11, Intake of Foods and Nutrients Among Adults in Sweden]. Uppsala: National Food Agency; 2012; (in Swedish).

[34] Bondonno CP, Liu AH, Croft KD, Ward NC, Shinde S, Moodley Y, Lundberg JO, Puddey IB, Woodman RJ, Hodgeson JM Absence of an effect of high nitrate intake from beetroot juice on blood pressure in treated hypertensive individuals: a randomized controlled trial. Am J Clin Nutr. 2015;102:368–75. 26135348 10.3945/ajcn.114.101188

[35] Carlström M, Liu M, Yang T, Zollbrecht C, Huang L, Peleli M, Borniquel S, Kishikawa H, Hezel M, Persson AE et al. Cross-talk between nitrate-nitrite-NO pathway and NO synthase pathways in control of vascular NO homeostasis. Antioxid Redox Signal. 2015;23:295–306. 24224525 10.1089/ars.2013.5481 PMC4523008

[36] Ashor AW, Lara J, Siervo M Medium-term effects of dietary nitrate supplementation on systolic and diastolic blood pressure in adults: a systematic review and meta-analysis. J Hypertens. 2017;35:1353–9. 28319596 10.1097/HJH.0000000000001305

[37] Montenegro MF, Sundqvist ML, Larsen FJ, Zhuge Z, Carlström M, Weitzberg E, Lundberg JO Blood pressure-lowering effect of orally ingested nitrite is abolished by a proton pump inhibitor. Hypertension. 2017;69:23–31. 27802417 10.1161/HYPERTENSIONAHA.116.08081

[38] Kapil V, Haydar SM, Pearl V, Lundberg JO, Weitzberg E, Ahluwalia A Physiological role for nitrate-reducing oral bacteria in blood pressure control. Free Radic Biol Med. 2013;55:93–100. 23183324 10.1016/j.freeradbiomed.2012.11.013 PMC3605573

[39] Bondonno CP, Liu AH, Croft KD, Considine MJ, Puddey IB, Woodman RJ, Hodgson JM Antibacterial mouthwash blunts oral nitrate reduction and increases blood pressure in treated hypertensive men and women. Am J Hypertens. 2015;28:572–5. 25359409 10.1093/ajh/hpu192

