# Supplementary material for: A randomized clinical trial of the effects of leafy green vegetables and inorganic nitrate on blood pressure
Source: Am J Clin Nutr. 2020 Feb 24;111(4):749–56. doi: 10.1093/ajcn/nqaa024 (PMC7338722; doi:10.1093/ajcn/nqaa024)
Supplement: nqaa024_Supplemental_File [file nqaa024_supplemental_file.docx]

**Online Supplementary Material**

**Supplementary Table 1. Inclusion and exclusion criteria.**

| **Inclusion criteria** |
| --- |
| Age 50-70 y |
| Clinic systolic blood pressure 130-159 mm Hg |
| Clinic diastolic blood pressure < 110 mm Hg |
| **Exclusion criteria** |
| Unwillingness or inability to modify current diet |
| Weight change > 5 % 3 months prior to study |
| Body mass index > 35 |
| Vegan or vegetarian |
| Use of antiseptic mouthwash |
| **Diseases** |
| Atrial fibrillation |
| Cardiovascular disease event the past 6 months |
| Malignancy or cancer therapy the past 6 months |
| Renal insufficiency |
| Rheumatologic disease |
| Inflammatory bowel disease |
| Hepatitis |
| Polycytemia Vera |
| Other chronic disease that may interfere with the effect of the study or with participation |
| **Medication** |
| Altered dose of anti-hypertensive medication the past 2 months |
| Insulin treated diabetes |
| Use of proton pump inhibitors |
| Use of organic nitrates |
| Use of antibiotics |
| Use of anticoagulants, Vitamin K inhibitors |
| Use of medicine for gout |
| Use of medicine for alcohol addiction |
| Use of medicine for erectile dysfunction |

**Online Supplementary Material**

**Supplementary Table 2. Laboratory data. Plasma levels of glucose, electrolytes and blood lipids.**

Groups

Placebo (n=78) Potassium nitrate (n=77) Leafy Green Vegetables (n=76)

Pre Post Pre Post Pre Post

Glucose, mmol/L

5.6 ±0.6 5.7 ±0.6^1^ 5.8 ±0.5 5.8 ±0.5 5.8 ±0.8 5.7 ±0.8

Sodium, mmol/L

141.3±1.8 140.9±2.8 141.2±1.8 141.2±2.3 140.7±2.1 140.4±2.4

Potassium, mmol/L

3.9±0.3 3.9±0.3 3.9±0.3 3.9±0.3 3.9±0.4 3.9±0.3

Magnesium, mmol/L

0.85±0.05 0.85±0.05 0.85±0.05 0.85±0.05 0.85±0.06 0.84±0.05

Phosphate, mmol/L

0.99±0.18 0.99±0.19 0.95±0.19 0.96±0.17 1.0±0.21 1.0±0.17

Iron, µmol/L

19.6±6.0 20.4±7.2 18.8±5.1 19.3±6.6 19.3±6.3 19.0±5.7

Calcium, mmol/L

2.4±0.08 2.4±0.07 2.4±0.08 2.4±0.08 2.4±0.09 2.3±0.09^1^

Cholesterol, mmol/L

5.7±0.8 5.7±0.8 5.8±1.0 5.9±1.0 5.8±1.2 5.7±1.2

Triglycerides, mmol/L

1.1±0.5 1.1±0.6 1.3±0.6 1.3±0.6 1.3±0.8 1.2±0.8

All values are means ±SDs.

^1^Significantly different from pre value p < 0.05

**Online Supplementary Material**

Supplementary Figure 1. Post intervention effects on plasma nitrate (A), plasma nitrite (B), saliva nitrate (C) and saliva nitrite (D). Concentration of urinary nitrate (E) and amount of excreted nitrate after a 24h urinary collection (F).

**Online Supplementary Material**


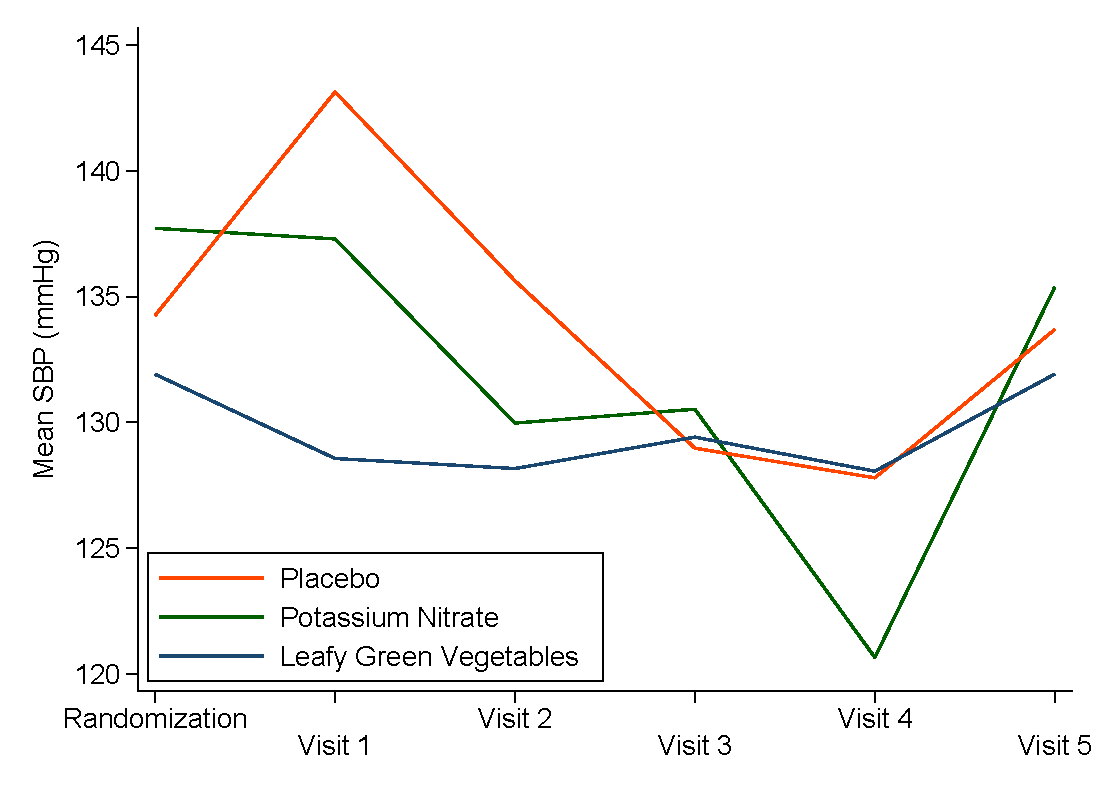
Supplementary Figure 2. Weekly systolic blood pressure during the 5 weeks of intervention analysed with Linear Mixed Models. Random-intercept model with crude time trajectories showed equal trajectories across the three groups, p = 0.3408.

**Online Supplementary Material**

**Supplementary Table 3. Random-intercept model with crude time trajectories for weekly systolic blood pressure.**

------------------------------------------------------------------------------

y | Coef. Std. Err. z P>|z| [95% Conf. Interval]

-------------+----------------------------------------------------------------

group |

B | -3.464369 5.141847 -0.67 0.500 -13.5422 6.613467

C | -5.806306 5.175348 -1.12 0.262 -15.9498 4.33719

|

visit |

4 | -.4220779 5.057343 -0.08 0.933 -10.33429 9.490131

5 | -7.746753 5.057343 -1.53 0.126 -17.65896 2.165456

6 | -7.194805 5.057343 -1.42 0.155 -17.10701 2.717404

7 | -17.07143 5.057343 -3.38 0.001 -26.98364 -7.159219

8 | -2.337662 5.057343 -0.46 0.644 -12.24987 7.574547

|

group#visit |

B#4 | 9.313104 7.129202 1.31 0.191 -4.659876 23.28608

B#5 | 9.124958 7.129202 1.28 0.201 -4.848021 23.09794

B#6 | 1.912754 7.129202 0.27 0.788 -12.06023 15.88573

B#7 | 10.60989 7.129202 1.49 0.137 -3.363089 24.58287

B#8 | 1.77997 7.129202 0.25 0.803 -12.19301 15.75295

C#4 | -2.926606 7.175651 -0.41 0.683 -16.99062 11.13741

C#5 | 3.996753 7.175651 0.56 0.578 -10.06726 18.06077

C#6 | 4.694805 7.175651 0.65 0.513 -9.369212 18.75882

C#7 | 13.21617 7.175651 1.84 0.066 -.8478515 27.28018

C#8 | 2.344241 7.175651 0.33 0.744 -11.71978 16.40826

|

_cons | 137.7208 3.647545 37.76 0.000 130.5717 144.8698

------------------------------------------------------------------------------

( 1) 2.group - 3.group = 0

------------------------------------------------------------------------------

y | Coef. Std. Err. z P>|z| [95% Conf. Interval]

-------------+----------------------------------------------------------------

(1) | 2.341937 5.158842 0.45 0.650 -7.769209 12.45308

------------------------------------------------------------------------------

( 1) 2.group#4.visit = 0

( 2) 2.group#5.visit = 0

( 3) 2.group#6.visit = 0

( 4) 2.group#7.visit = 0

( 5) 2.group#8.visit = 0

( 6) 3.group#4.visit = 0

( 7) 3.group#5.visit = 0

( 8) 3.group#6.visit = 0

( 9) 3.group#7.visit = 0

(10) 3.group#8.visit = 0

chi2( 10) = 11.22

Prob > chi2 = 0.3408

**Online Supplementary Material**

Supplementary Figure 3. Flow mediation dilation pre and post 5 weeks of intervention was unchanged in all groups (p> 0.05).
